# Successful Management of a Rare and Severe Community-Acquired Pyopneumothorax Complicated by Refractory Sepsis: A Case Report

**DOI:** 10.7759/cureus.101270

**Published:** 2026-01-10

**Authors:** Rongzong Cai, Xinping Wang, Shunda Yuan

**Affiliations:** 1 Department of Emergency Clinic, Xiamen Changgung Hospital, Xiamen, CHN; 2 Department of Critical Care Medicine, Xiamen Changgung Hospital, Xiamen, CHN; 3 Department of Thoracic Surgery, Xiamen Changgung Hospital, Xiamen, CHN

**Keywords:** case report, hypernatremia, pyopneumothorax, refractory infection, sepsis

## Abstract

Community-acquired pyopneumothorax progressing to refractory sepsis is a rare but life-threatening condition. This report details the successful management of a 62-year-old male who presented with septic shock and acute respiratory failure secondary to a massive right-sided pyopneumothorax. The case was notable for an extreme inflammatory response and the development of persistent hypernatremia. Management in the intensive care unit involved urgent thoracic drainage, broad-spectrum antibiotics, and meticulous supportive care over a two-month period, ultimately leading to the patient's recovery. This case highlights the complexities of managing such severe infections and underscores the critical importance of early diagnosis, source control, and the management of complex electrolyte imbalances like hypernatremia in the septic patient. The complete longitudinal data provide valuable insight into the dynamic progression and resolution of this critical illness.

## Introduction

Pyopneumothorax, the presence of both pus and air in the pleural space, is a serious complication often arising from pneumonia or lung abscess. While less common in the modern antibiotic era, it can rapidly progress to sepsis and septic shock, carrying a high mortality rate (approximately 15%), with 30% of cases requiring early surgical intervention such as pleural drainage [1]. The pathophysiology involves a severe systemic inflammatory response to infection, which can lead to multi-organ dysfunction. A particularly challenging complication in this setting is the development of hypernatremia, which is associated with increased mortality and reflects significant disturbances in water homeostasis, often exacerbated by the critical illness itself [2]. This case report presents a rare and severe instance of community-acquired pyopneumothorax, with a focus on its successful management and the unique challenge of correcting concomitant refractory hypernatremia, adding to the limited literature on the management of such complex cases.

## Case presentation

A 62-year-old male with no significant past medical history presented to the emergency department with a three-day history of acute dyspnea, high-grade fever, and right-sided pleuritic chest pain. On admission, the patient was critically ill, with a temperature of 39.0°C, heart rate of 135 beats per minute, respiratory rate of 35 breaths per minute, blood pressure of 85/50 mmHg, and an oxygen saturation of 85% on room air. Physical examination revealed altered mental status, absent breath sounds, and dullness to percussion over the right hemithorax.

**Figure 1:**
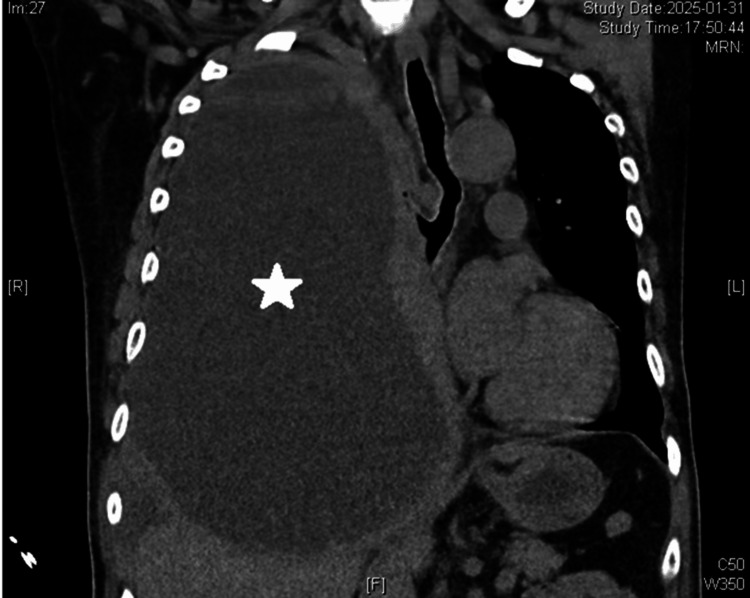
Admission Chest CT Scan Coronal chest images demonstrate a large right-sided pyopneumothorax (asterisk). The mediastinal window confirms extensive pleural effusion.

Initial laboratory studies were significant, as summarized in Table 1. Key abnormalities included marked leukocytosis, significantly elevated inflammatory markers, and severe hypernatremia.​ Arterial blood gas analysis revealed severe metabolic acidosis (pH 7.17). A subsequent emergency chest computed tomography (CT) scan was obtained for definitive diagnosis. The CT imaging revealed a large right-sided pyopneumothorax with complete lung collapse (Figure 1), a finding critical for diagnosis and management planning. Areas of primary diagnostic interest are indicated by digitally produced arrows.

**Table 1:** Laboratory Parameters on Admission

Parameter	Patient Value	Normal Range	Interpretation
White Blood Cell Count (WBC)	26.72x10^9^/L	3.5-9.5x10^9^/L	Marked leukocytosis, indicative of severe infection.
High-Sensitivity C-reactive Protein (HS-CRP)	357.90mg/L	0-5mg/L	Extreme elevation, suggesting significant systemic inflammation.
Procalcitonin (PCT)	>50ng/mL	<0.05ng/mL	Highly elevated, consistent with bacterial sepsis.
Serum Sodium	160mmol/L	135-145mmol/L	Severe hypernatremia.

The patient was immediately intubated for respiratory failure and transferred to the ICU. Volume-controlled mechanical ventilation was initiated. Due to weaning failure, a tracheostomy was performed on February 10, 2025, and mechanical ventilation was continued via the tracheostomy tube. Weaning trials began on February 27, 2025, with successful liberation from ventilation and transfer to the general ward on March 3, 2025.​ A right-sided thoracic closed drainage (tube thoracostomy) was emergently performed, resulting in the initial drainage of over 800 mL of foul-smelling pus.

**Figure 2:**
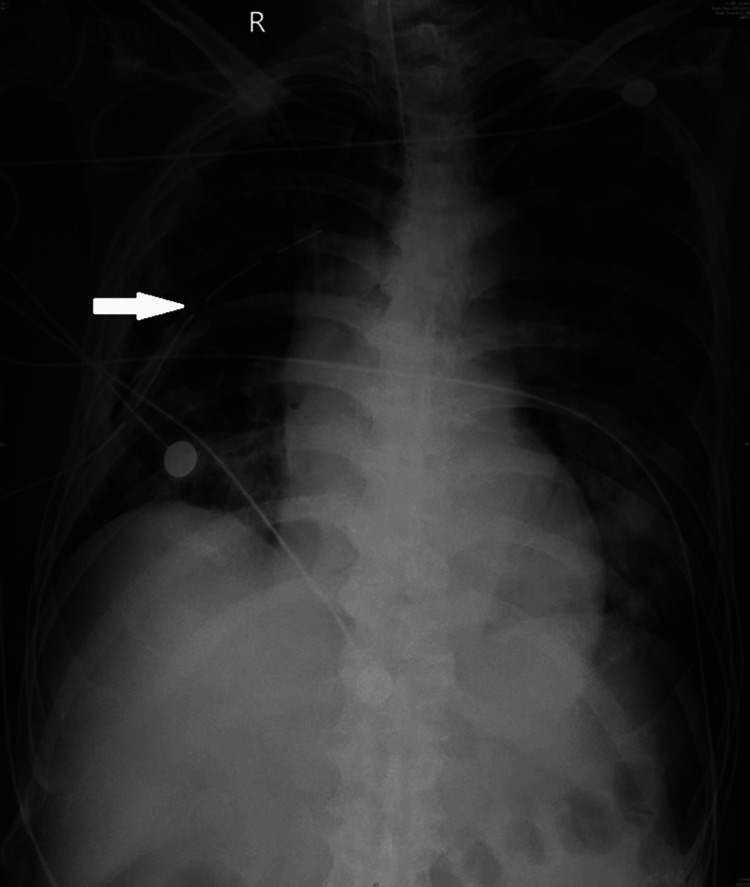
Mid-treatment Portable Chest X-ray An anteroposterior view shows the right-sided thoracic drainage tube in situ (white arrow). There is interval reduction in the right pleural opacity compared with the initial CT scan, consistent with partial resolution of the pyopneumothorax.

**Figure 3:**
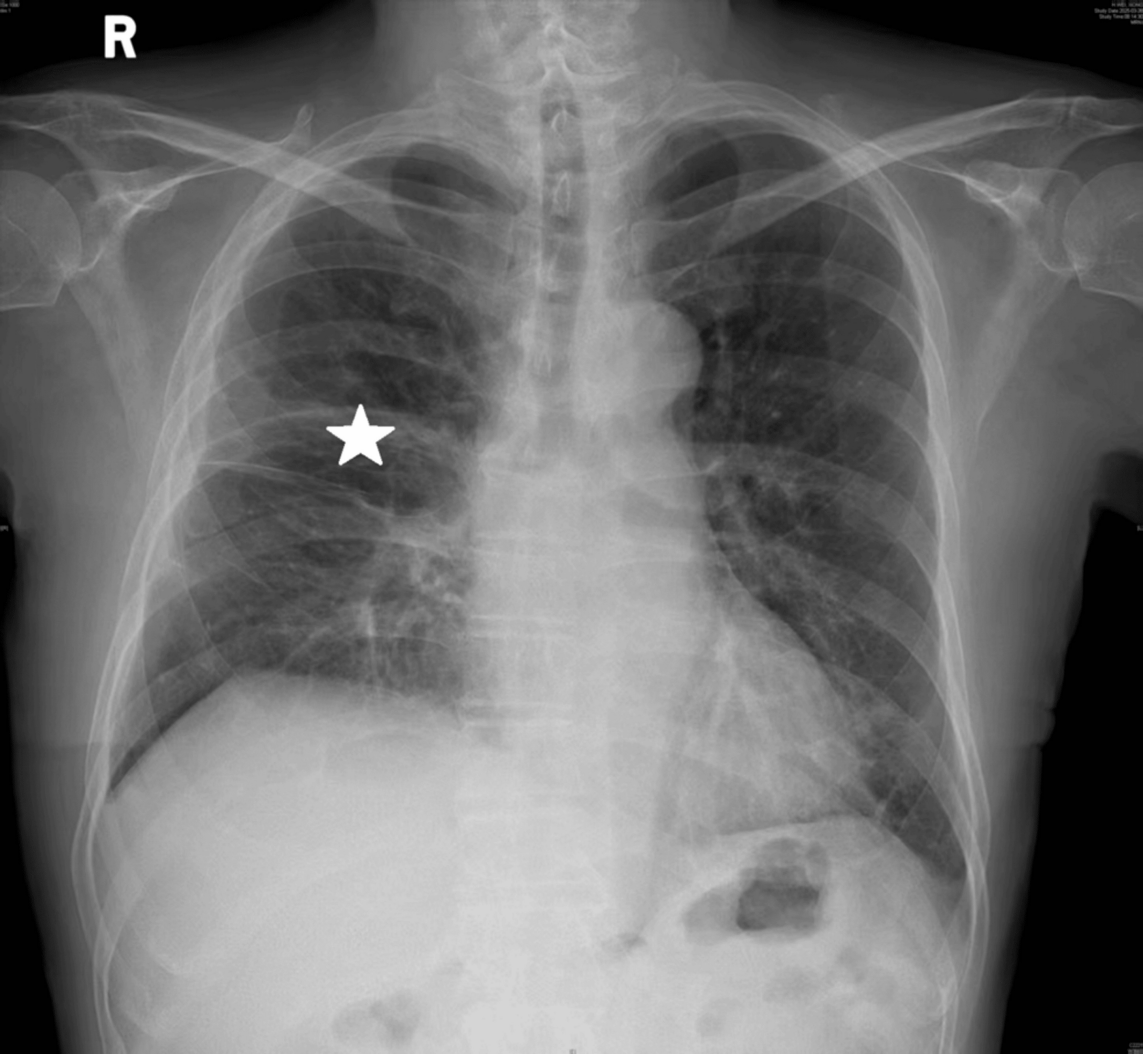
Pre-discharge Chest X-ray A portable chest radiograph reveals near-complete resolution of the right pyopneumothorax and satisfactory re-expansion of the right lung (asterisk).

The antimicrobial regimen was initiated with meropenem (1 g intravenously every eight hours), vancomycin (1 g intravenously every 12 hours), and metronidazole (0.5 g intravenously every eight hours). Vancomycin was discontinued on February 6, 2025, following clinical improvement. On February 11, 2025, therapy was de-escalated to penicillin G (4.8 million units intravenously every six hours) after significant clinical improvement.​ The patient required vasopressor support for septic shock. The clinical course was prolonged and complicated by persistently elevated sodium levels, requiring careful fluid and electrolyte management. A series of chest X-rays was taken during the hospitalization to monitor progress. A mid-treatment X-ray obtained showed the in-situ chest tube with partial resolution of the opacity (Figure 2), while a final X-ray prior to discharge demonstrated significant clearing and satisfactory lung re-expansion (Figure 3). After a total hospitalization of approximately 55 days, the patient was discharged in stable condition.

## Discussion

This case illustrates a severe presentation of community-acquired pyopneumothorax leading to refractory sepsis and marked hypernatremia. The successful outcome was likely attributable to several key factors, most importantly the rapid diagnosis via CT imaging and the immediate source control achieved through tube thoracostomy. The management of this patient aligns with the fundamental principle in sepsis treatment: timely and effective infection source control is paramount [3].

**Figure 4:**
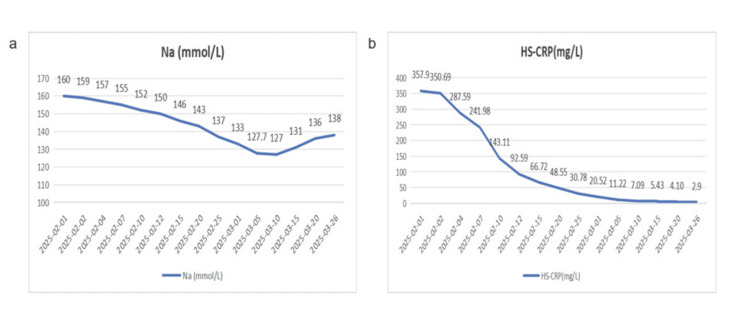
Trend of Key Laboratory Parameters This figure shows the gradual correction of refractory hypernatremia (Na^+^) (a) and the rapid decline in high-sensitivity C-reactive protein (hs-CRP) (b) over time.

A notable feature of this case was the persistent hypernatremia (Figure 4a), which proved to be a major management challenge [2]. The hypernatremia was considered refractory due to its persistence despite standard fluid management. Its development was likely multifactorial, contributing to its refractory nature, including the systemic inflammatory state impairing renal water conservation and possible insensible losses.​ In critically ill septic patients, hypernatremia is a recognized prognostic factor, with ICU-acquired hypernatremia (IAH) being linked to chronic critical illness (CCI) and persistent inflammation, immunosuppression, and catabolism syndrome (PICS) [4]. The trend of the patient's sodium levels, along with the inflammatory marker CRP (Figures 4a, 4b), showing the slow resolution of hypernatremia paralleling the control of infection, underscores this association. The meticulous management of hypernatremia, involving sodium restriction and careful free water replacement, was a critical component of the supportive care.

The patient's protracted course in the ICU highlights the phenomenon of sepsis-induced immunosuppression. The extended need for supportive care may reflect this complex immunopathology. When compared with existing literature, this case is distinguished by the combination of the rarity of community-acquired pyopneumothorax, its progression to septic shock, and the prominent challenge of refractory hypernatremia. The serial radiographic and laboratory data provide a comprehensive view of the disease's evolution. This complete dataset offers a valuable educational resource for understanding the natural history and management response of such a critical illness. Our treatment approach, including early broad-spectrum antibiotics and source control, was in accordance with the Surviving Sepsis Campaign guidelines for the management of severe sepsis [3].

## Conclusions

This report demonstrates that a multidisciplinary approach emphasizing early radiographic diagnosis, prompt source control, appropriate antimicrobial therapy, and meticulous supportive care, including the management of complex electrolyte disturbances like hypernatremia, can lead to a successful outcome even in cases of rare and severe community-acquired pyopneumothorax with septic shock. The case underscores the importance of recognizing and managing the complications of sepsis, such as persistent hypernatremia and potential immunosuppression, which significantly impact patient recovery. Further research into personalized immunomodulatory therapies may improve outcomes for patients with similar severe septic presentations.
